# Goblet Cells Contribute to Ocular Surface Immune Tolerance—Implications for Dry Eye Disease

**DOI:** 10.3390/ijms18050978

**Published:** 2017-05-05

**Authors:** Flavia L. Barbosa, Yangyan Xiao, Fang Bian, Terry G. Coursey, Byung Yi Ko, Hans Clevers, Cintia S. de Paiva, Stephen C. Pflugfelder

**Affiliations:** 1 Department of Ophthalmology, Baylor College of Medicine, Houston, TX 77030, USA; Flaleao10@gmail.com (F.L.B.); Yangyan.xiao@bcm.edu (Y.X.); bftongji@hotmail.com (F.B.); terrygcoursey@gmail.com (T.G.C.); kopupil@hanmail.net (B.Y.K.); cintiadp@bcm.edu (C.S.d.P.); 2 Department of Ophthalmology, the Second Xiangya Hospital, Central South University, Changsha 410011, China; 3 Department of Ophthalmology, Konyang University Hospital, College of Medicine, Konyang University, Daejeon 302-718, Korea; 4 Hubrecht Institute, 3584 CT Utrecht, The Netherlands; h.clevers@hubrecht.eu

**Keywords:** mucins, antigen, goblet cell, dendritic cells, adaptive immunity, immune tolerance

## Abstract

Conjunctival goblet cell (GC) loss in dry eye is associated with ocular surface inflammation. This study investigated if conjunctival GCs contribute to ocular surface immune tolerance. Antigens applied to the ocular surface, imaged by confocal microscopy, passed into the conjunctival stroma through goblet cell associated passages (GAPs) in wild type C57BL/6 (WT), while ovalbumin (OVA) was retained in the epithelium of SAM pointed domain containing ETS transcription factor (*Spdef*) knockout mice (*Spdef*^−^/^−^) that lack GCs and are a novel model of dry eye. Stimulated GC degranulation increased antigen binding to GC mucins. Induction of tolerance to topically applied OVA measured by cutaneous delayed type hypersensitivity (DTH) was observed in WT, but not *Spdef*^−^/^−^. OTII CD4^+^ T cells primed by dendritic cells (DCs) from the conjunctival draining lymph nodes of *Spdef*^−^/^−^ had greater IFN-γ production and lower Foxp3 positivity than those primed by WT DCs. These findings indicate that conjunctival GCs contribute to ocular surface immune tolerance by modulating antigen distribution and antigen specific immune response. GC loss may contribute to the abrogation of ocular surface immune tolerance that is observed in dry eye.

## 1. Introduction

Gel-forming mucins secreted by goblet cells (GCs), through their ability to retain water, can form a highly hydrated mucus gel that covers and protects mucosal surfaces [1]. Conjunctival GCs secrete mucins that stabilize the tear layer and lubricate and protect the surface of the eye [2]. Mucins have the capability to trap allergens and debris and clear them from mucosal surfaces. However, this function has not been thoroughly evaluated on the ocular surface. Conjunctival GC loss occurs in aqueous tear deficient dry eye and certain ocular surface inflammatory diseases, including Sjögren syndrome (SS), Stevens-Johnson syndrome, ocular mucous membrane pemphigoid, and graft-versus-host-disease [3,4,5]. GC loss is associated with greater ocular surface epithelial disease and higher expression of the Th1 cytokine IFN-γ in aqueous deficient dry eye and mouse models of dry eye, suggesting that conjunctival GC mucins have an immunomodulatory function [5,6,7]. Mice null for the SAM pointed domain containing ETS transcription factor gene (*Spdef*^−^/^−^) lack GCs and have been found to have a dry eye phenotype with corneal barrier disruption, conjunctival inflammatory cell infiltration, and increased expression of inflammatory cytokines in the conjunctiva [8]. *Spdef* has been found to have immunomodulatory functions. Overexpression of Spdef in airway epithelium of neonatal mice promoted inflammatory cell infiltration and increased expression of chemokines and cytokines that mediate Th2 inflammation, including CCL24, interleukin 33 (IL-33), and IL-13 [9]. Furthermore, the Th2 response to house dust mite (HDM) extract induced pulmonary inflammation was significantly attenuated in the *Spdef*^−^/^−^ strain [9].

Goblet cell associated passages (GAPs) under cholinergic regulation have been described in the mouse small intestine [10,11]. These GAPs deliver luminal antigens to a subset of dendritic cells (DCs) in the underlying lamina propria that are known to promote immune tolerance [10]. The conjunctiva covers two-thirds of the ocular surface and contains an abundant GC population. As the most exposed mucosal tissue in the body, the ocular surface is constantly exposed to self and foreign antigens in the tears. Antigens topically applied to the conjunctiva have been found to induce immune tolerance [12,13], but the role of conjunctival GCs in regulating antigen distribution and induction of immune tolerance has not been investigated. It is hypothesized that conjunctival GCs regulate antigen passage to the stroma and promote tolerogenic properties in DCs as a mechanism to maintain immune tolerance on the ocular surface. This study explored the function of GCs in distribution of topically applied antigen and induction of tolerance to antigens applied to the ocular surface of wild type and *Spdef*^−^/^−^ mice.

## 2. Results

### 2.1. Goblet Cells and Distribution of Antigens Topically Applied to the Conjunctiva

**Figure 1:**
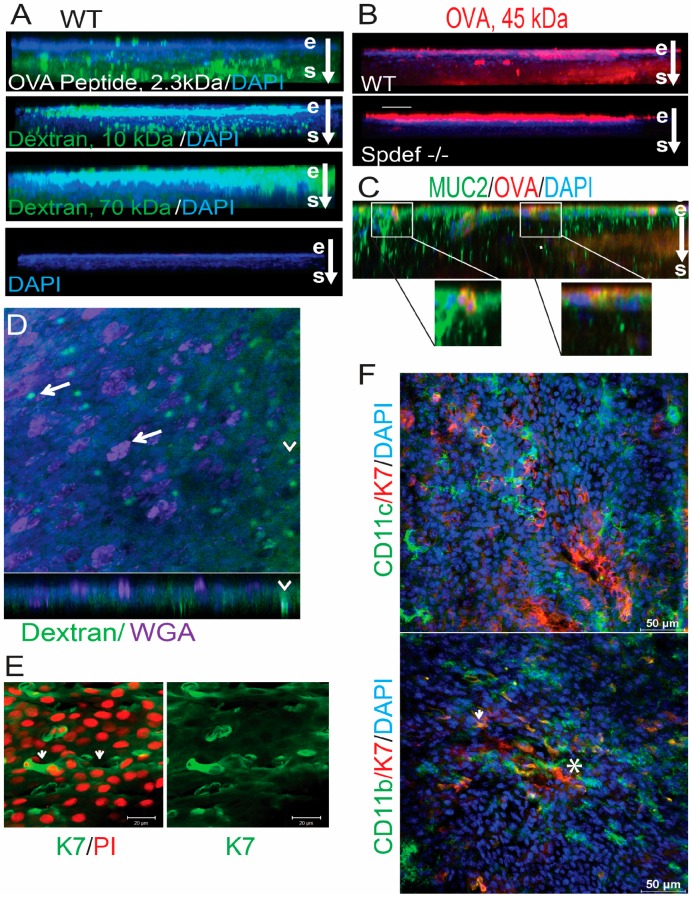
Goblet cell associated passages (GAPs) are present in the conjunctiva. Representative laser confocal microscopy images of whole mount conjunctivas taken from six mice for each antigen (*n* = 6/group) are shown. (**A**) Z-stack option from epithelium (e) to stroma (s) showing distribution of three different sized antigens 30 min after topical application to the ocular surface. Nuclei are stained with DAPI. Top to bottom—the greatest stromal passage was noted with OVA peptide 2.3 kDa (green) and Dextran 10 kDa (green) that bound to the surface epithelium and migrated in a columnar pattern into the stroma. Dextran 70 kDa (green) localized primarily to the surface epithelium, but columns extending into the superficial stroma were also noted. Negative control with 4′,6-diamidino-2-phenylindole (DAPI, blue) nuclear stain, but no antigen instilled is shown at the bottom; (**B**) 45 kDa OVA (red) was noted in the epithelium and in columns in the stroma of WT (top), but was localized to the epithelium only in the *Spdef*^−^/^−^ (bottom) (*n* = 6); (**C**) Z-stack option from epithelium to stroma showing distribution of MUC2^+^ (green) and OVA^+^ (red) cells in WT mice. White squares demarcate goblet cells with co-localization of MUC2 and OVA that are shown at higher magnification below; (**D**) Top—surface of whole mount conjunctiva stained with WGA lectin (purple) that bound to goblet cell glycoproteins and Dextran 10 kDa (green). Minimal dextran binding was noted in WGA^+^ filled goblet cells (arrows), whereas dextran was noted to pass into and through empty goblet cells (WGA negative) into the underlying stroma (arrowheads indicate dextran filled goblet cells with an underlying column of dextran stromal fluorescence in Z-stack); (**E**) Surface of whole mount conjunctiva stained for goblet cell marker keratin 7 (in green) and nuclei in red with goblet cell openings marked with arrows. Scale bar is 20 µm; (**F**) Whole mount conjunctivas stained for cytokeratin 7 (K7, red) and CD11c (top, green) or CD11b c (bottom, green) cells with nuclei stained with DAPI (blue). Some CD11b^+^ cells had a dendritic morphology (asterisk) and others stained positively for K7 (arrow), *n* = 6 per antigen.

This study evaluated the distribution of fluorescent antigens topically applied to the mouse conjunctiva. (Figure 1A). Antigens, including 2.3 kDa OVA peptide, and 10 and 70 kDa dextrans, passed into the stroma through goblet cell associated passages (GAPs) in WT (Figure 1A). The least epithelial retention was noted for OVA peptide, and the greatest stromal localization was noted for the two smallest sized antigens, 2.3 kDa OVA peptide and 10 kDa dextran which migrated in columnar or oval patterns. There was less stromal diffusion of 70 kDa dextran than 10 kDa dextran, but columns were noted in the stroma for both antigens (Figure 1A). No GC staining was noted when unconjugated ovalbumin (not shown) or no antigen was placed on the eye (Figure 1A, bottom). Ovalbumin (OVA, 45 kDa), the antigen used in our immune tolerance assays also passed into the stroma in WT, but it was retained within the epithelium in the *Spdef*^−^/^−^ (Figure 1B). The observed columnar pattern of antigen passage into the stroma in WT suggests they migrated through the GCs in a pattern similar to what has been observed in the GAPs in the small intestinal mucosa where the greatest passage through GCs was observed with smaller antigens as we also observed in the conjunctiva [11]. The co-localization of the GC mucin MUC2 and OVA in Figure 1C confirms that antigens were passing through GAPs in normal conjunctiva. In contrast, OVA antigen was primarily retained on and in the epithelium of *Spdef*^−^/^−^ strain that lacks GCs.

As further evidence that antigens were passing into the stroma through open GCs, we labeled the GCs in whole mount conjunctivas from WT mice with the lectin wheat germ agglutinin (WGA) after instilling 10 kDa dextran onto the ocular surface pre-mortem. WGA lectin binds to *N*-acetyl glucosamine and *N*-acetyl neuraminic acid in the GC glycoconjungates and was found to have high affinity for apical cells of the corneal and conjunctival epithelium and for conjunctival GCs in light/fluorescent microscopic studies [14,15]. Dextran co-localized with WGA in some filled GCs (arrows), but showed greater migration into and passage through empty GCs (arrowheads) (Figure 1D), suggesting there is greater antigen passage into the stroma through empty GCs. These circular openings in the conjunctiva were confirmed to be GCs by immunostaining with the GC specific cytokeratin 7 (K7, Figure 1E).

Similar to other mucosal tissues, the conjunctiva contains environmentally sensing DCs [16]. We found two populations of DCs in the conjunctiva of normal WT mice, both in close proximity to GCs. CD11c^+^ cells in the epithelium have a morphology resembling Langerhan’s cells with the dendrites of some cells surrounding the openings of cytokeratin 7 (K7) positive GCs (Figure 1F, top). Plumper CD11b^+^ cells with either an oval or shorter dendritic morphology (asterisk) were primarily located in the basal epithelium and underlying stroma and some of these cells also abutted on GCs and stained positively for K7 (arrow, Figure 1F, bottom).

### 2.2. Modulation of Antigen Distribution by Cholinergic Stimulation

**Figure 2:**
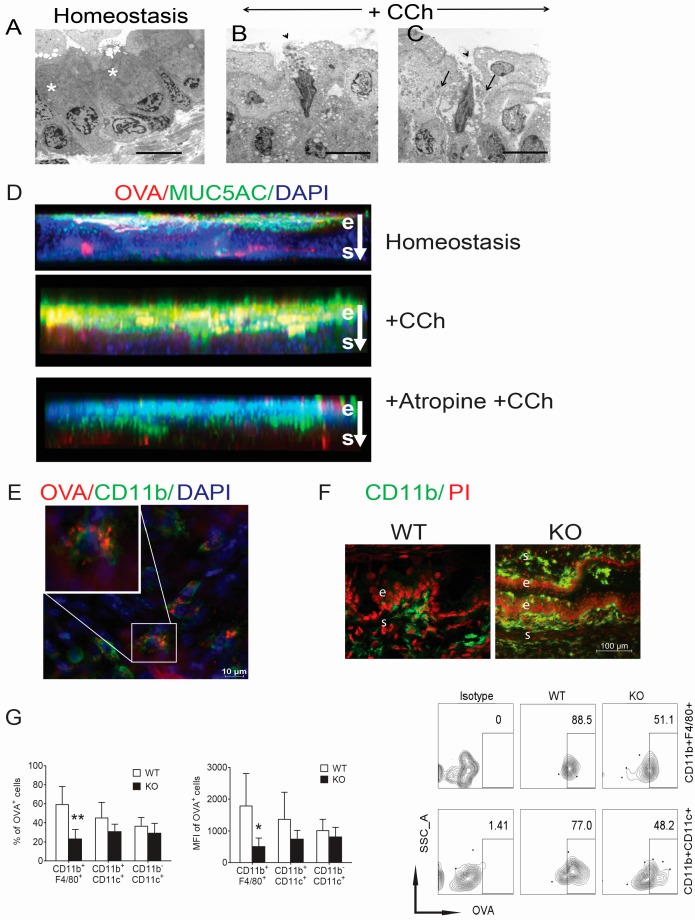
Goblet cells under cholinergic regulation serve as antigen conduits. (**A**–**C**) Transmission electron microscopy of conjunctival goblet cells. (**A**) In homeostasis, goblet cells are filled with mucus granules (asterisks); (**B**) 20 min after stimulation with cholinergic agonist, carbachol choline (CCh), granules in goblet cell openings appear smaller in size and darker (arrowhead); (**C**) Passages within and around some discharged goblet cells were observed (arrows). Magnification: 3000×. Scale bar 4 µm (in **A**–**C**); *n* = 4 per group; (**D**) Representative images of whole mount conjunctivas stained for MUC5AC. Mice received either OVA drops 30 min prior to euthanasia (homeostasis), cholinergic stimulation 20 min prior to OVA drops (+CCh), or atropine 30 min prior to CCh and OVA (+Atropine +CCh). Atropine blockade prior to cholinergic stimulation limited OVA passage into GCs and stroma, *n* = 3; (**E**) Whole mount conjunctiva stained for CD11b (green) 30 min after topical administration of OVA (red) with nuclei stained with DAPI (blue). White square demarcates OVA^+^CD11b^+^ cell that is shown in higher magnification at the upper right; (**F**) In conjunctival tissue sections, CD11b^+^ cells were found just below the basal conjunctival epithelium in WT (left), while they were present on the surface of the epithelium and within the epithelium and stoma in the *Spdef*^−^/^−^ (KO, right); (**G**) Left. Mean ± SD of flow cytometry analysis of OVA^+^ antigen presenting cells 4 h after topical administration of OVA_23–339_ peptide on the ocular surface of WT C57BL/6 and *Spdef*^−^/^−^ (KO) strains. Frequency and mean fluorescence intensity (MFI) of OVA^+^ cells are shown (*n* = 6 per strain); * *p* < 0.05; ** *p* < 0.01; comparison WT vs. *Spdef*^−^/^−^ Right. Representative dot plots of flow cytometry analysis showing uptake of OVA_323–339_ peptide (OVA) after topical administration. Conjunctivas were harvested, collagenase-digested and single cell suspensions were stained with CD11b, CD11c and F4/80 antibodies.

Conjunctival GCs have been found to secrete in response to cholinergic agonists, such as carbamylcholine (CCh) [17] Transmission electron microscopy was performed to gain a better understanding of changes in GC morphology, release of mucin containing secretory granules, and opening of GAPs in the conjunctiva following CCh stimulation and representative electron micrographs are shown in Figure 2A–C. GCs contained intact mucus granules under homeostatic conditions (asterisks, Figure 2A). Exocytosis was detected after topical CCh stimulation (arrowhead, Figure 2B). Additionally, there was a change in the GC morphology with elongation of the nucleus and development of channels within and around some GCs (arrows, Figure 2C). Conjunctival GCs produce and secrete the gel-forming mucins MUC2 and MUC5AC. The effects of stimulated mucin secretion on distribution of topically applied OVA were examined, an antigen that has been widely used to characterize antigen uptake, processing, and presentation by dendritic cells and in studies of adaptive immunity [13]. In homeostasis, topically applied OVA bound the conjunctival surface and was noted in MUC5AC-filled conjunctival GCs and the underlying stroma (Figure 2D, top). Following CCh stimulation, there was greater OVA localization to MUC5AC throughout the epithelium (e) and GCs, as well as the underlying stroma (Figure 2D, middle). Treatment with the muscarinic receptor antagonist atropine prior to CCh inhibited migration of OVA into the GCs and underlying stroma (Figure 2D, bottom). These findings indicate that antigen binding to GC secreted mucins and passage through GAPs in the conjunctiva is under cholinergic regulation.

To determine if GAPs serve as conduits for antigens to adjacent antigen presenting cells, this study investigated if topically applied FITC-conjugated OVA_323–339_ peptide was phagocytosed by the macrophage or dendritic cells located adjacent to the GCs. The greatest antigen uptake was noted in CD11b^+^ cells that are located at the base of the GCs in the WT (Figure 2E,F). In contrast CD11b^+^ cells are located on and within the epithelium and stroma of the *Spdef*^−^/^−^ (KO, Figure 2F). Flow cytometry was used to measure the relative uptake of OVA peptide by these cell populations after topical application in WT and *Spdef*^−^/^−^ mice (Figure 2G). There was a greater percentage and mean fluorescent intensity (MFI) of OVA peptide^+^ CD11b^+^F4/80^+^ macrophages and CD11b^+^CD11c^+^ DCs in the WT conjunctiva than the *Spdef*^−^/^−^ that reached significance (*p* < 0.05) in CD11b^+^ F4/80^+^ cells (Figure 2G, left). A representative scatter plot of OVA peptide^+^ cells in the two populations is shown in Figure 2G, right. These suggest indicate that GAPs in the conjunctiva serve as conduits for antigen migration into the stroma and to the adjacent phagocytic immune cells, particularly the CD11b^+^ F4/80^+^ macrophages.

### 2.3. Lack of Goblet Cells Abrogates Induction of Conjunctival Immune Tolerance

**Figure 3:**
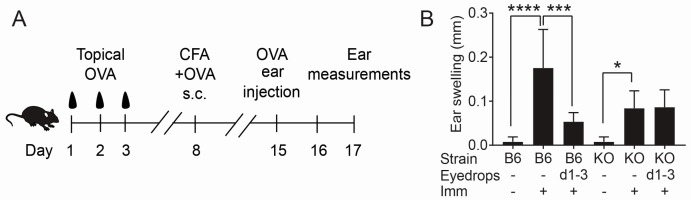
Goblet cell loss in *Spdef*^−^/^−^ abrogates induction of conjunctival immune tolerance. (**A**) Conjunctival immune tolerance was measured by delayed type hypersensitivity (DTH) to ovalbumin (OVA). Mice with or without pre application of OVA drops for three consecutive days received S.C. immunization (Imm) with OVA + complete Freund’s adjuvant (CFA) on day 8 and were challenged with OVA antigen by intradermal ear injection (OVA right ear and PBS left ear) on day 15. Ear swelling was measured after 48 h. Naïve mice without immunization served as controls; (**B**) In vivo DTH assay (ear swelling) measured 48 h after challenge. Results are means ± SD of ear swelling after calculating the difference between the antigen-injected and PBS-injected ears for each mouse (*n* = 5 animals/group). Induction of conjunctival tolerance was not observed in *Spdef*^−^/^−^ (KO). Imm = immunization of OVA + CFA; B6 = C57BL/6 wild-type; KO = *Spdef*^−^/^−^; d = days. * *p* < 0.05; *** *p* < 0.001, **** *p* < 0.0001 comparison WT vs. KO.

It is well recognized that immune tolerance develops to antigens such as OVA that are topically applied to the ocular mucosal surface [12,18,19,20]. Soluble factors produced by the conjunctiva were found to condition tolerogenic properties in DCs [18]. Experimental dry eye and treatment of the ocular surface with the preservative benzalkonium chloride have been reported to inhibit tolerance to topically applied OVA and both are associated with GC loss [18,19]. Based on these findings and previous reports that immunomodulatory factors are produced by cultured conjunctival GCs [15,21], it was hypothesized that conjunctival GCs are essential for inducing ocular surface immune tolerance. To address this issue, the ability to induce conjunctival immune tolerance was compared to topically applied OVA antigen in WT and *Spdef*^−^/^−^ strains by assessing cutaneous delayed type hypersensitivity (DTH) to OVA after mice were immunized with complete Freund’s adjuvant (CFA) mixed with OVA. A separate group of WT and *Spdef*^−^/^−^ mice received OVA eye drops for three consecutive days a week before they were immunized. Ear challenge was performed a week after the systemic immunization. The protocol is summarized in Figure 3A. Immunization with OVA elicited significant ear swelling in both WT and *Spdef*^−^/^−^ groups compared to naïve, non-immunized mice, although the magnitude of the response in *Spdef*^−^/^−^ was lower than WT (Figure 3B). As previously reported [12,18], it was observed that conjunctival tolerance was induced in WT mice that received topical OVA eye drops prior to immunization compared to immunized control mice that did not receive drops. In contrast, no induction of conjunctival immune tolerance was observed in the *Spdef*^−^/^−^ strain (Figure 3B), indicating that GC contribute to tolerance induction in the conjunctiva.

**Figure 4:**
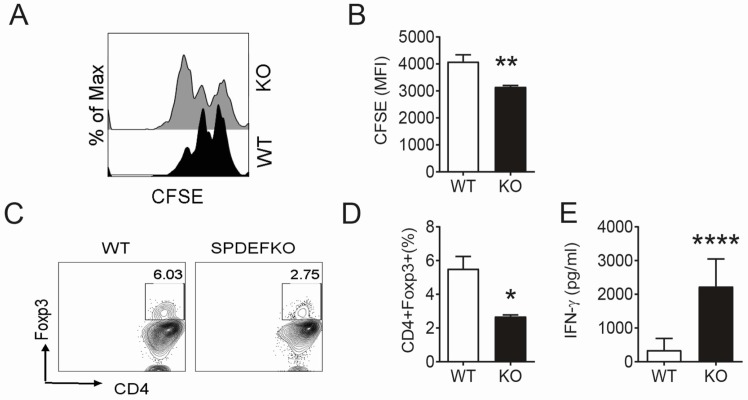
*Spdef*^−^/^−^ dendritic cells (DCs) prime Th1 cells. Cervical lymph node (CLN) cell suspensions of from either B6 WT or *Spdef*^−^/^−^ mice (KO, *n* = 4 group) were pulsed with OVA_323–339_ peptide and co-cultured with CD4^+^ T cells isolated from OT II mice for 4–5 days. Cells were collected for measuring proliferation and IFN-γ was measured in supernatants by immunobead assays. Data shown in (**A**–**E**) are from representative experiments. (**A**) Representative CFSE histograms showing fluorescence dilution (measure of proliferation) in OT II cells after priming with either WT or KO CLN suspensions; (**B**) Mean fluorescence intensity (MFI) of CFSE^+^CD4^+^ cells. Means ± SD, *n* = 4 wells/group; (**C**) Representative dot-plots of CD4^+^Foxp3^+^ used to generate graph in D; (**D**) CD4^+^Foxp3^+^ percentage in the two groups. Means ± SD, *n* = 4 wells/group; (**E**) IFN-γ concentration in supernatants of mixed lymphocyte reactions measured by Luminex assay. Means ± SD, *n* = 4 wells/group. Statistical tests, Student *t* test comparison WT vs. SPDEFKO. * *p* < 0.05; ** *p* < 0.01; **** *p* < 0.001.

To investigate the basis for the lack of tolerance induction in the *Spdef*^−^/^−^ strain, this study compared the ability of antigen presenting cells (APCs) in the conjunctival draining cervical lymph nodes (CLN) of WT and *Spdef*^−^/^−^ mice to induce antigen specific lymphoproliferation, generate CD4^+^ Foxp3^+^ cells (a marker of regulatory T cells) and stimulate IFN-γ production (Figure 4). Single cell CLN suspensions from WT or *Spdef*^−^/^−^ mice were pulsed with OVA_323–339_ peptide and co-cultured with OT II CD4^+^ T cells for 3–4 days. *Spdef*^−^/^−^ APCs stimulated greater proliferation (Figure 4A,B) and suppressed Foxp3 expression in the OTII CD4^+^ cells (Figure 4C,D). Additionally, IFN-γ production was significantly greater in OTII CD4^+^ T cells primed with APCs from *Spdef*^−^/^−^ (Figure 4E). These findings suggest that conjunctival GCs have a tolerizing effect on APCs in the draining nodes.

## 3. Discussion

The purpose of this study was to evaluate the effects of GCs on distribution, mucin binding, and induction of immune tolerance to antigens topically applied to the mouse conjunctiva. It was found that during homeostasis, antigens bound GC mucins in the conjunctiva and passed into the stroma through GAPs, with greatest observed migration with smaller molecules. Electron microscopy showed passages opened within (on either side of the elongated nuclei) and around GCs following cholinergic induced secretion. The finding that topically applied dextran passed into and through non-filled GCs, suggests that one function of homeostatic GC mucin secretion is to maintain GAPs. Stimulated GC secretion also increased antigen binding to GC mucin on the conjunctival surface. Our findings suggest there are similarities in antigen binding and GC passage between the conjunctiva and the small intestine. In both tissues, GC mucins bind topically applied antigens and GAPs serve as conduits for antigen passage to the stroma, including the resident phagocytic immune cells [10]. The size disparity between GC openings shown in whole mount conjunctiva by confocal microscopy in Figure 1D and transmission electron microscopy (EM) in Figure 2A may be attributed to the fixation and embedding protocol for electron microscopy and that confocal imaged openings of GC clusters on the surface, while EM images showed cross sections of single GCs.

The finding that topically applied antigen to the conjunctiva induces immune tolerance is consistent with previously reported studies [12,18,19,20]. Galletti and associates found that induction of tolerance was disrupted in eyes with experimental dry eye or that received benzalkonium chloride (BAC) [18]. Additionally, these studies found that soluble factors from whole conjunctival explants suppressed lymphoproliferation in mixed lymphocyte reactions. These studies did not evaluate the specific role of GCs in tolerance induction. The findings of this study suggest that GCs have a key function in induction of conjunctival immune tolerance. This may be due to multiple factors, including directed antigen passage through GAPs toward antigen presenting cells and lymphatics in the conjunctival stroma, antigen binding and clearance by GC mucins and production of factors that tolerize DCs. It appears that the CD11b^+^ cells located at the base of the GCs can sample GC content because some of these cells contained the GC specific cytokeratin 7. Lamina propria CD11b^+^ dendritic cells adjacent to GCs in the small intestine have been found to contain GC specific cytokeratin 18 [10]. Additional studies will be required to determine the relative contribution of these CD11b^+^ cells in maintaining conjunctival immune tolerance.

Loss or dysfunction of conjunctival GCs is a well-recognized pathologic feature of aqueous deficient dry eye and certain ocular surface inflammatory conditions, including Stevens-Johnson syndrome and graft-vs.-host disease [3,4,5]. We reported that IFN-γ expression in the conjunctiva was significantly higher in eyes with GC loss and was found to be inversely correlated with conjunctival GC density [5]. IFN-γ has also been reported to disrupt cholinergic signaling and secretory function in GCs and approximately 80% of patients with Sjögren syndrome, a systemic autoimmune disease that causes severe dry eye, develop anti-M3R autoantibodies that can also disrupt cholinergic signaling [22,23]. DC maturation and migration to the draining cervical lymph nodes was found by our group to be an initiating event in the induction of the Th1 and Th17 response in our experimental desiccating stress mouse model of dry eye that is induced by suppression of tear production by systemic cholinergic blockade and exposure to a desiccating environment [6,24,25,26]. Using the same dry eye model, Galletti and colleagues reported that tolerance to OVA antigen applied to the conjunctiva is lost when the antigen administration is started after three days of systemic anti-cholinergic treatment [19]. This study suggests that the conjunctival GCs have a tolerizing effect on DCs in the draining nodes because CLN cell suspensions from *Spdef*^−^/^−^ stimulated greater proliferation and IFN-γ production and lower Foxp3 expression in antigen primed CD4^+^ T cells in vitro.

In summary, this study confirmed the hypothesis that GCs modulate antigen distribution and induction of immune tolerance in the conjunctiva. Cholinergic stimulated GC secretion resulted in GAP opening and greater antigen binding by mucins. GC loss abrogated induction of conjunctival immune tolerance. These findings suggest that OS inflammation in dry eye could be related to altered tolerance to self-antigens in the tears or from cells residing on the ocular surface or to exogenous antigens (e.g., microbes or allergens). This work provides justification for further investigation in this area that might provide insight into strategies to modulate the ocular surface immune response and to protect the ocular surface against damaging inflammation in conditions associated with GC loss or dysfunction.

## 4. Materials and Methods

### 4.1. Reagents

Antibodies used for immunofluorescent staining included (antigen/catalog no./concentration): MUC2/15334/200 µg/mL and MUC5AC/16903/200 µg/mL (Santa Cruz Biotechnology, San Diego, CA, USA), CD11b/550282/125 µg/mL and CD11c/553799/0.5 µg/mL (BD Biosciences, San Jose, CA, USA) and cytokeratin 7/ab9021/200 µg/mL (Abcam, Cambridge, MA, USA). Secondary antibodies (goat anti-rabbit and hamster and donkey anti-mouse and goat), ovalbumin Alexafluor^®^ 488 (O34784), Dextran 10 kDa (D7170) and 70 kDa Oregon Green (D7173) were from Thermofisher, Atlanta, GA, USA. Goat anti-rat IgG was from Jackson ImmunoResearch Laboratories, West Grove, PA, USA. OVA_323–339_ peptide FITC (1 mg; KP1304) was from KareBayBiochem, Monmouth Junction, NJ, USA.

### 4.2. Mice

Female C57BL/6 (*n* = 83) and B6.Cg-Tg(TcraTcrb)425Cbn/J (OT-II, *n* = 15) mice 6–8 weeks old were purchased from the Jackson Laboratory (Bar Harbor, ME, USA). *Spdef*^−^/^−^ mice (*n* = 25) were obtained from Jeffrey Whittsett and Hans Clevers and were bred in a conventional specific pathogen free vivarium. The Institutional Animal Care and Use Committees at Baylor College of Medicine approved all animal experiments (Animal protocol AN-2032, approved dates 4-1-15 to 3-31-18). All studies adhered to the Association for Research in Vision and Ophthalmology statement for the Use of Animals in Ophthalmic and Vision Research.

### 4.3. In vivo Administration of Fluorescent Antigens

A 2.5 µL drop of fluorescent antigens (OVA, OVA peptide or Dextran 10 and 70 kDa (50 µL/mL) was applied in each/eye. The animals were euthanized by isoflurane/cervical dislocation (CD) 30 min later. Whole-mount freshly harvested conjunctivas were fixed with either cold acetone (antigens and dendritic cell markers) or cold methanol (WGA lectin, anti-keratin 7, MUC2 and MUC5AC) for 10 min. After fixation, they were washed in PBS for 10 min. The tissues were blocked with 1% BSA for 1 h to reduce nonspecific labeling. Primary antibodies diluted 1:50 in 1% BSA were applied for 1 h at room temperature (RT). Tissues were washed with 1% PBS, then incubated with appropriate secondary antibodies diluted 1:250 in PBS for 60 min at RT in the dark, then counterstained with DNA binding Hoechst 33342 or propidium idodide dyes diluted 1:500 for 10 min. After washing with PBS, conjunctivas were flattened on slides and coverslips mounted with Gel/Mount (Thermofisher, Atlanta, GA, USA). Unconjugated OVA and no antigen were used as positive and negative controls, respectively. Distribution of each fluorescent antigen was evaluated in conjunctival whole mounts from 6 separate mice (*n* = 6 per antigen).

### 4.4. Wheat Germ Agglutinin (WGA) Staining

A 2.5 µL drop of dextran 10 kDa was applied in each/eye. The animals were euthanized by isoflurane/CD after 30 min. Whole-mount freshly harvested conjunctivas were fixed in cold methanol for 10 min. After fixation, they were washed in PBS for 10 min and then incubated with WGA diluted 1:200 in PBS for 60 min at RT in a dark room. After 1 h, they were washed in PBS for 10 min and counterstained with DNA binding Hoechst dye diluted 1:500 for 10 min. After washing with PBS, conjunctivas were flattened on slides and coverslips applied with Gel/Mount. Co-localization of dextran and WGA was evaluated in conjunctival whole mounts from 6 mice (*n* = 6).

### 4.5. Cholinergic Stimulation of Goblet Cell Secretion

GC secretion was stimulated by applying 2.5 µL of the pan-muscarinic acetylcholine receptor (AchR) agonist carbamylcholine chloride 0.1 mM (CCh, Sigma, C4382) to the surface of each eye 20 min before antigen application based on a report that this concentration stimulated secretion by rat conjunctival GCs [27]. This study initially evaluated CCh concentrations of 0.01 and 0.1 mM and found a greater percentage of secreting goblet cells with 0.1 mM CCh by confocal microscopy. Consequently, we used only 0.1 mM for the experiments we report. The pan muscarinic receptor antagonist atropine (2.5 µL of 1% solution) was applied to the conjunctiva 30 min prior to CCh stimulation. Experiments evaluating effects of cholinergic stimulation were performed in four control and for CCh treated eyes (*n* = 4 per group). Mice were euthanized by isoflurane/CD 30 min after antigen application and whole-mount freshly harvested conjunctivas were fixed in cold acetone for 10 min. After fixation, they were washed in PBS for 10 min.

### 4.6. Immunostaining and Laser Scanning Confocal Scanning Microscopy

Tissues were blocked with 1% BSA for 1 h to reduce nonspecific labeling. Primary antibodies against CD11c or CD11b diluted 1:50 in 1% BSA were applied for 1 h at RT. Tissues were washed with 1% PBS for 1 h, then appropriate secondary antibodies diluted 1:250 in PBS were applied for 60 min at RT in the dark. This was repeated with anti-K7, then counterstained with Hoechst dye for 10 min. After washing with PBS, conjunctivas (*n* = 3 animals/group) were flattened on slides and coverslips mounted with Gel/Mount. Digital confocal images were captured with a laser scanning confocal microscope (Nikon A1 RMP, Nikon, Melville, NY, USA) wavelength 400–750 nm and 1 µm Z-step. The images were processed using NIS Elements 4.20 version (Nikon).

### 4.7. Transmission Electron Microscopy

Conjunctivas from four eyes with or without CCh stimulation 20 min prior were fixed in 3% glutaraldehyde, washed in 0.1 M sodium phosphate buffer, pH 7.3, post fixed in 1% osmium tetroxide and dehydrated in a series of graded alcohols. Tissues were infiltrated and hardened with acetone and PolyBed 812 plastic resin and embedded in plastic molds with 100% plastic resin. Thick (1 micron) and thin sections (80–90 nm) sections were cut on a Leica Ultracut R ultramicrotome (Buffalo Grove, IL, USA). Thick sections were placed on a glass slide and stained with toluidine blue. Thin sections were place on copper mesh grids and stained with uranyl acetate and lead citrate. Samples were viewed on a Zeiss EM902 transmission electron microscope (Thornwood, NY, USA) and images captured with a AMT V602 (Woburn, MA, USA) digital camera.

### 4.8. Detection of Topically Applied Antigens in the Conjunctiva

A topical dose of 2.5 µL of FITC-labeled OVA_323–339_ peptide (OVAp, 5 mg/mL, KareBay Biochem Inc, Monmouth Junction, NJ, USA) was topically instilled on each eye of WT and *Spedf*^−^/^−^ mice and animals were euthanized 4 h later (*n* = 6 for each strain). Conjunctivas were excised and incubated with 0.1% Collagenase IV (Invitrogen/ThermoFisher Scientific, Grand Island, NY, USA) and 0.05 mg/mL of DNAse I (EMD Millipore/Millipore Sigma) in a shaker at 37 °C for 1 h, followed by two sequential washes with complete RPMI. A single cell suspension was made by passing cells through a 70 µm cell strainer, and cells were centrifuged and resuspended for flow cytometry. Cell suspensions were stained with anti-CD16/32 (4 °C, 10 min), and were subsequently stained using anti-CD45_BV510 (clone 30F11, BD Biosciences, San Jose, CA, USA) anti-CD11c APC (clone HL3, BD Biosciences, San Jose, CA, USA), anti-F4/80_PE (clone BM8, Biolegend,San Diego, CA), anti-CD11b-PE-Cy7 (clone M1/70, BD Biosciences, San Jose, CA, USA) antibodies, resuspended in buffer containing live/dead fixable dye, and kept on ice until immediate analysis on a BD Canto II Benchtop cytometer with BD Diva software version 6.7 (BD Biosciences, San Jose, CA, USA). The following gating strategy was used in this study: dead cells were excluded by gating alive CD45^+^ cells, subsequently gated on the basis of forward scatter height versus forward scatter area (singlets 1), then gated on side scatter height versus side scatter area (singlets 2). CD11b and CD11c were then plotted. CD11c^−^CD11b^+^ cells were >90% positive for F4/80 antigen.

### 4.9. Cell Proliferation Assay

Antigen specific T cell proliferation was measured by mixing cervical lymph node (CLN) cell suspensions, OVA peptide and OTII CD4^+^ T cells. CLN cell suspensions from WT and *Spdef*^−^/^−^ mice (*n* = 4 mice per strain) were prepared by smashing nodes between two glass slides. Cells were counted, plated at 5 × 10^5^/500 µL per well and pulsed with OVA_323–339_ peptide (10 μg/mL, InvivoGen, San Diego, CA, USA) for 1 h. CD4^+^ T cells were isolated from 6–8 week old female B6.Cg-Tg(TcraTcrb) 425Cbn/J (OT-II) OT-II mice according to the manufacturer’s instructions (untouched CD4^+^ T cell isolation kit, (StemCell technologies, Cambridge, MA, USA), CFSE labeled (Thermofisher, Grand Island, NY, USA), plated at 5 × 10^5^/500 µL per well in 96-well plates and incubated for 3–4 days. Cells were collected and used for flow cytometry while supernatants were collected and stored at −80 °C until use. These experiments were performed two times.

### 4.10. Flow Cytometry Analysis

For intracellular Foxp3 staining, primed OT-II single cell suspensions (*n* = 4 wells/group) were stained with infra-red fluorescent reactive dye (Thermofisher, Grand Island, NY, USA) for 30 min prior to incubation with Foxp3 Fixation/Permeabilization working solution (eBioscience/ThermoScientific, Waltham, MA, USA). Cells were washed with 1× Permeabilization solution and incubated with anti-CD16/32, followed by staining with anti-CD4-APC (clone GK1.5, BD Bioscience, San Jose, CA, USA), and anti-Foxp3-APC (clone FJK-16S, eBioscience/ThermoScientific, Waltham, MA, USA), washed and resuspended. The cells were kept on ice until flow cytometry analysis was performed. The gating strategy used in this study was as follows: dead cells were excluded by gating infra-red dye negative cells, subsequently gated on the basis of forward scatter height versus forward scatter area (singlets 1), then gated on side scatter height versus side scatter area (singlets 2). Cells were then gated on CD4^+^ cells and frequency of CD4^+^Foxp3^+^ was recorded. Negative controls consisted of fluorescence minus one (FMO) splenocytes. A BD Canto II Benchtop cytometer was used for flow cytometry, and data were analyzed with BD Diva software version 6.7 (BD Biosciences, San Jose, CA, USA) and FlowJo software version 10. (Tree Star Inc., Ashland, OR, USA). This experiment was performed two times.

### 4.11. Conjunctival Immune Tolerance and DTH Assay

Conjunctival immune tolerance was measured by cutaneous delayed type hypersensitivity (DTH) to ovalbumin (OVA) using the following protocol: OVA eyedrops (5 µL/eye of 2 mg/mL solution) were administered topically for three days in both WT and KO groups (*n* = 5/group). On day 8, mice received immunization with an emulsion of OVA + complete Freund’s adjuvant (Thermofisher, Grand Island, NY, USA) prepared 1:1, subcutaneously in the flanks under general anesthesia (100 µg/animal). On day 15, mice were challenged with antigen by intradermal ear injection (10 µg of OVA right ear and PBS left eye). Ear swelling was measured after 24 and 48 h using a gauge micrometer (Mitotoyo, Japan). Results show ear swelling as measured by the difference between the antigen-injected and PBS injected ears for each mouse. A group of naïve unimmunized mice and a group of immunized mice without OVA drops served as controls.

### 4.12. Cytokine Immunobead Assay

Culture assay supernatants (from three wells for each experimental group) were collected and cytokine production was determined by an immunobead assay. Samples were added to wells containing the appropriate cytokine bead mixture that included mouse monoclonal antibody specific for IFN-γ (Upstate-Millipore, Billerica, MA, USA). Serial dilutions of IFN-γ were added to wells in the same plate as the culture supernatants to generate a standard curve. The plates were incubated overnight at 4 °C to capture the cytokine by the antibody conjugated fluorescent beads. After three washes with assay buffer, 25 µL of biotinylated secondary cytokine antibody mixture was applied for 1.5 h in the dark at room temperature. The reactions were detected with streptavidin–phycoerythrin using a Luminex 100 IS 2.3 system (Austin, TX, USA). The limit of detection of this assay was 3.4 pg/mL for IFN-γ.

### 4.13. Statistical Analysis

Between group mean differences between WT and *Spdef*^−^/^−^ KO mice were compared by Student *t* test using Prism 6.0 (GraphPad, San Diego, CA, USA). Sample sizes were calculated to have ≥90% power of detecting significant between group differences (α 0.05) based on anticipated results.

## 5. Conclusions

Conjunctival goblet cells serve as conduits for antigen transport to immune cells in the underlying stroma. Immune tolerance to antigens topically applied to the conjunctiva is lost in mice lacking goblet cells. These findings indicate that conjunctival goblet cells function to maintain immune tolerance on the ocular surface. These findings may have implications for dry eye conditions with goblet cell loss where immune tolerance is disrupted.
